# Reviews and Book Notices

**Published:** 1880-12

**Authors:** 


### Reviews and Book Notices.

---

ARTICLE IX.—A HANDBOOK OF PHYSICAL DIAGNOSIS, COMPRISING THE THROAT, THORAX AND ABDOMEN. By Dr. Paul Guttman, Privat Docent, University of Berlin. Translated from the third German edition.

In this valuable work, the condensed result of a long clinical experience in the University of Berlin, the author presents the various methods of clinical examination of the thoracic and abdominal viscera, in health and disease. As far as the nature of each subject permits, he observes uniform order in its arrangement as to details, taking up first the signs obtained by each method of exploration, then their causes, and the physiological and pathological conditions in which they occur. This work forms a compact volume of 344 pages, including an appendix on laryngoscopy. It has been much noticed abroad, has passed through a half-dozen translations in different languages. Wood's Library Standard Medical Authors, 1880. D.

ARTICLE X.—NAVAL HYGIENE: HUMAN HEALTH AND THE MEANS OF PREVENTING DISEASE. With illustrative incidents, principally derived from naval experience. By Joseph Wilson, M.D., Medical Director U. S. Navy. Second edition, with colored lithographs. Philadelphia: Lindsay & Blakiston; 1879.

As time goes on, the subject of hygiene is gradually coming to the front and taking the place in medical education to which it is entitled. The National Board of Health, the various State Boards of Health, and, under their supervision, numerous village and city organizations of a like character, are all working toward the same end. "*How to prevent*" is quite as important as "How to cure." Among the excellent treatises upon hygiene, this book of Dr. Wilson's takes a high place. The fairness of statement which characterizes the author's writings, as well as the practical information conveyed by them, help to make this volume a valuable book of reference to the physician. While it is especially devoted to the consideration of the "Hygiene of Ships," and to the discussion of the best means of preventing disease among sailors and seafaring men, it is yet of value to the general reader.

Chap. XXIII, which considers the important subject of "Yellow Fever," is quite too short, and condensed to a space which is not commensurate with the importance of this terrible scourge to (at least) American seamen. A more extended article, giving additional information upon the hygiene of this disease, would make the book much more valuable. Taken as a whole, the work is an excellent one, and ought to be read by every physician. B. W. G.

Article XI.—Physicians' Combined Call Book and Tablet, and Physicians' Handy Ledger. Published by Ralph Walsh, M.D., Washington City, D. C. Price, $3.00 and $1.50.

We have been using these two books in our professional work for some time past, and find them the most convenient in size, shape and system we have ever used. By them any physician, no matter how extensive his practice, can by a few minutes' attention keep a detailed account of his services. The ledger well deserves its title "handy," for the simplicity and accuracy of its method is very marked. B.

Article XII.—Lessons in Gynæcology. By Wm. Goodall, A.M., M.D., Professor of Clinical Gynæcology in the University of Pennsylvania. Second edition.

Many of these lectures are well known to the profession through their appearance, from time to time, in various medical journals throughout the country; and their collection into this interesting volume has been no doubt duly appreciated. The reader will hardly tire of the book before the last page is finished. Admirably clear in style and eminently practical, theory has little space, but energetic, painstaking work fills each hour. The reader sits in the amphitheatre, the patient is brought in, and the entertainment proceeds.

Chapters XXX, "On Nerve Tire and Womb Ills," and XXXI, "On Special and General Hints for the Prevention of Uterine Disorders," are practical hygienic sermons from life.

We commend the work to the profession especially, as adapted to the thorough commitment to memory by the younger practitioners. D.

ARTICLE XIII.—A NOTE ON THE ALKALOIDS OF CINCHONA. By BENJ. LEE, M.D., PH. D., etc. Read before the Medical Society of the State of Pennsylvania in May, 1880.

This is a short paper, the chief object of which appears to be the showing of how many active principles the cinchona bark contains, and to advertise the combination of several of these in the form known as Quinquinia. The writer enumerates eighteen primary and seven secondary distinct active principles as having been obtained from the bark. They are named as follows:

NATURAL ALKALOIDS.

| *Crystallizable.* | *Amorphous.* |
|---|---|
| Quinia. | Dihomocinchonia. |
| Quinidia. | Paytamia. |
| Cinchonia. | Cusconidia. |
| Cinchonidia. | Dicinchonia. |
| Quinamia. | Diquinidia. |
| Quinidamia. | Paricia. |
| Homocinchonia. | A liquid alkaloid unmamed. |
| Homocinchonidia. | |
| Paytia. Cusconia. | |
| Aricia. Jauania. | |

*Artificial or Secondary Alkaloids.*

Produced from the crystalline alkaloids by overheating or by certain chemical reactions.

| | |
|---|---|
| Quinicia. | Quinamicia. |
| Cinchonicia. | Homocinchonicia. |
| Quinamidia. | Apoquinamia. |
| Protoquinamicia. | |

